# Exploring the structure and properties of $\alpha$-sheet based bilayer borophenes

**DOI:** 10.1038/s41598-024-82972-w

**Published:** 2025-01-02

**Authors:** Subrata Rakshit, Nevill Gonzalez Szwacki

**Affiliations:** Faculty of Physics, University of Warsaw, Pasteura 5, 02093 Warsaw, Poland

**Keywords:** Bilayer borophene, Electronic transport, DFT, Electronic properties and materials, Two-dimensional materials, Computational nanotechnology

## Abstract

Recent experimental realizations of bilayer boron materials motivated us to study the structure and properties of $\alpha$-sheet-based bilayer borophenes with interlayer covalent bonds. As shown here, at least three stacking variations are possible: AA, AB, and $\hbox {AB}'$. The on-top AA-stacking has been obtained experimentally supported on a metallic substrate. The AB-stacking is the most stable among neutral freestanding structures, whereas the AA and $\hbox {AB}'$ stacking sequences are very close in energy, both for neutral and negatively charged cases. The studied bilayer borophenes exhibit extraordinarily high electric conductivity with values as high as ${\sim } 10^7\mathrm {~S}/\textrm{m}$ for the experimentally observed AA-stacking. The highly stable AB-stacking bilayer, reported here for the first time, exhibits an anisotropic conductivity with an average value of $6.0 \times 10^6~\mathrm {~S}/\textrm{m}$. Contrary to the AA-stacking bilayer that retains the 6-fold fold rotational symmetry of the $\alpha$-sheet, the AB-stacking structure has 2-fold symmetry, which leads to the anisotropic transport properties.

## Introduction

Boron is a prototypical electron-deficient element whose structures display unique allotropic forms not seen in other periodic table elements. The diversity of structures reported experimentally and theoretically for bulk boron is even larger for structures with smaller dimensions^1^. The investigation of extended 2D boron structures intensified after the theoretical reports for the stability of the hollow $\hbox {B}_{{80}}$ boron cluster^2^ and the structurally related $\alpha$-sheet^3^. Later on, the $C_{6v}$-$\hbox {B}_{{36}}$ quasi-planar cluster with a central hexagonal hole has been successfully synthesized on an Ag(111) substrate^4^. This structure can be seen as the precursor of the $\alpha$-sheet, and it is the first experimental confirmation of the existence of 2D boron allotropes named borophenes. Although the $\alpha$-sheet is the most stable form of a one-atom-thick boron layer, it tends to buckle^5^. Therefore, it is not as stable as graphene, and its exfoliation from a substrate may be a challenge^6^. In addition, the electronic deficiency of boron atoms leads to complex bonds that, in turn, give rise to various borophene polymorphs. These different phases are defined by different hexagonal hole arrangements and the corresponding hole concentration, $\eta$. The most interesting borophene phases include but are not limited to $\alpha ~(\eta =1/9)$, $\beta _{12}~(\eta =1/6)$, and $\chi _3~ (\eta =1/5)$^ 7^.

Bilayer (BL) borophene comprises two atomic-layer-thick sheets bonded together with some space between them. The interacting layers reinforce each other in a BL form of 2D boron, leading to a more stable structure^5,8^. Several theoretical and experimental works have been done on BL structures based on different borophene allotropes^5,8–11^. However, several questions remain to be answered. For example, early theoretical studies have suggested that BL borophene’s interlayer distance is 2.5–3 Å, suggesting a van der Waals (vdW) interaction between the layers^5^. However, synthesized BL borophenes show a much closer interlayer distance, around 2 Å, implying relatively strong covalent bonds.

The BL structures were recently synthesized by the MBE method on $\textrm{Ag}(111), \textrm{Ru}(0001)$, and $\textrm{Cu}(111)$ substrates and were found to possess remarkable conductivity and greater stability than the monolayer counterparts^8,12,13^. From that group of experiments, the work focused on the controlled boron deposition on atomically flat single-crystal Ag(111)^8^ is of our special interest. In that work, by comparing bond-resolved experimental images with first-principles calculations, the atomic structure of the observed BL borophene was consistent with two covalently bonded $\alpha$-sheets. The synthesized structures were several nanometers long and were labeled as BL-$\alpha$ borophenes. They were shown to preserve the metallicity of the $\alpha$-sheet but with higher structural order and 6-fold rotation symmetry. Calculations have shown that the substrate donates electrons, and the charge transfer is predominantly localized on the bottom layer^8^. More recently, the BL-$\alpha$ borophene was shown to be less sensitive to ambient conditions than the boron monolayer structures, bringing closer the not yet achieved ex-situ characterization^14^. The synthesis of multilayer structures^15,16^ is another route for realizing novel boron structures possibly less affected by the substrate^17^.

The BL (and multilayer) borophenes could be used for energy or chemical storage. For instance, there are theoretical predictions that BL borophene is a promising material for batteries since having space between the layers provides a place to hold lithium ions^18^. The BL borophene has also emerged as a promising topological material, exhibiting unique semimetal properties and Fermi velocities comparable to graphene ones. Density functional theory (DFT) has been used to demonstrate that BL borophene is a Dirac material characterized by a nodal line^19^.

Nevertheless, the experimental studies reported so far on BL borophenes are limited to a few structures only, and much more work needs to be done to understand the structural and electronic properties of BL and multilayer borophenes. Therefore, despite extensive theoretical and experimental studies, the most stable BL structure, both freestanding or supported on a substrate, has yet to be determined. This work presents the results of first-principles calculations for freestanding BL boron structures consisting of two $\alpha$-sheets. Different arrangements between the layers have been considered. For the most stable structures, the electronic properties are studied.

## Results and discussion

**Fig. 1:**
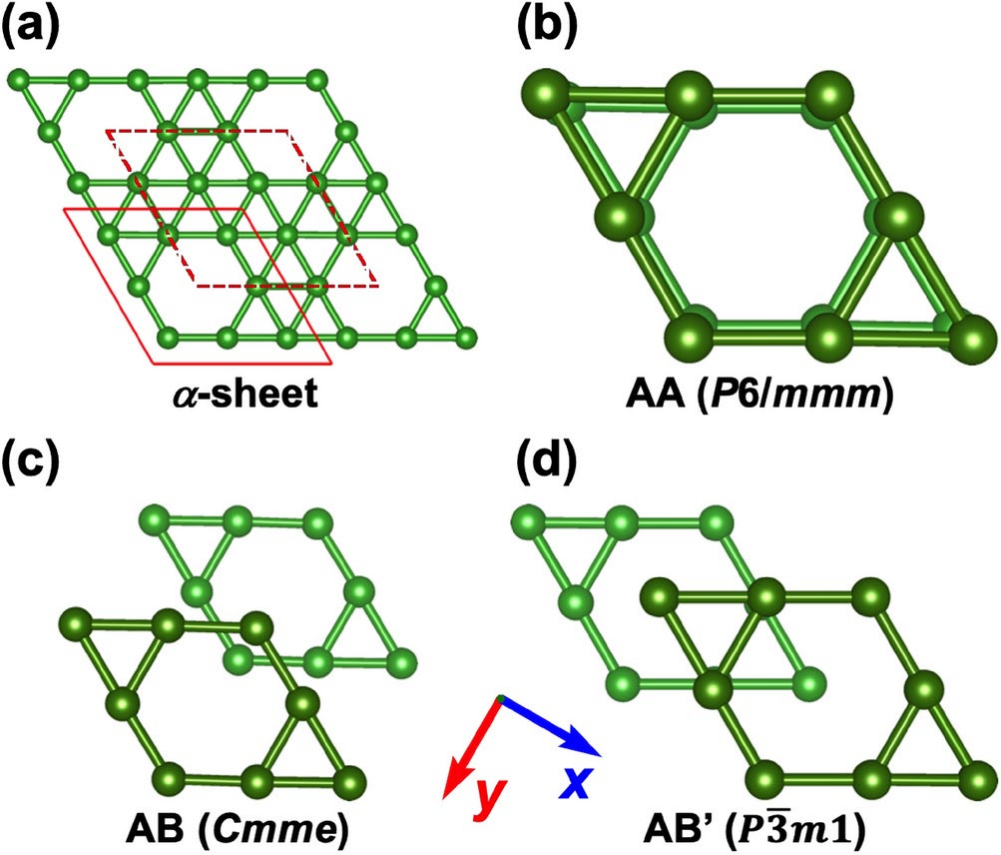
Three high-symmetry stacking sequences of $\alpha$-sheet-based BL borophenes considered in this study. (**a**) The structure of the $\alpha$-sheet with two unit cells highlighted in red. (**b**) The on-top AA-stacking which has been observed experimentally. (**c**, **d**) Two ways of constructing the AB-stacking: the top layer is shifted with respect to the bottom layer in the *y* and *x* directions in (**c**) and (**d**), respectively.

As mentioned above, the $\alpha$-sheet structure is slightly buckled^5^, and the deviation from planarity is responsible for the lowering in symmetry from *P*6/*mmm* to *C*2/*m*. The unit cell of the $\alpha$-sheet structure is shown in Fig. 1a and consists of 6 atoms forming a planar hexagon with 2 additional atoms attached to its parallel opposite edges. Those two “floppy” atoms have out-of-plane positions oriented towards opposite sides of the plane. As starting configurations, we have used fully planar $\alpha$-sheets forming three distinct high symmetry arrangements labeled as AA, AB, and $\hbox {AB}'$ shown in Fig. 1. The AA-stacking (Fig. 1b) is formed by two $\alpha$-sheets stacked directly on top of each other, forming a structure with *P*6/*mmm* symmetry. By shifting one of the layers with respect to the other in the *x* direction, we obtain the $\hbox {AB}'$-stacking (Fig. 1d) with $P {\overline{3}} m 1$ space symmetry. Doing a similar shift but in the *y* direction gives rise to the AB-stacking (Fig. 1c) with *Cmme* symmetry. We have, therefore, 6-, 3-, and 2-fold rotational symmetries for the AA, $\hbox {AB}'$, and AB structures, respectively. The $\hbox {AB}'$-stacking is similar to BL graphene, where the center of an empty hexagon in one layer is located on top of the center of the filled hexagon of the opposing layer. In contrast, in the AB-stacking, the rhombus marked with a solid red line in Fig. 1a of one layer is located on top of the rhombus marked with a dashed red line, shown in Fig. 1a, of the opposing layer.

**Fig. 2:**
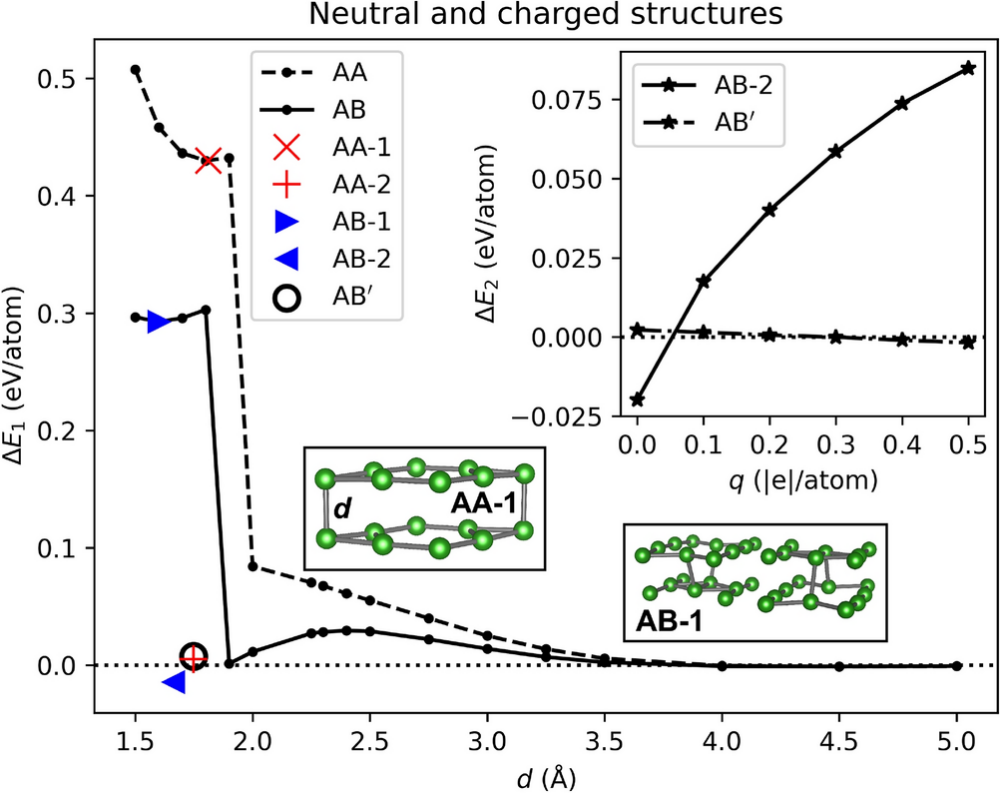
Difference between the energy of the BL borophene and the energies of the isolated $\alpha$-sheets. $\Delta E_1=E_t-E_{\alpha \text{-sheet }}$, where $E_t$ and $E_{\alpha \text{-sheet }}$ are the total energies per atom of the boron BL and the $\alpha$-sheet, respectively. In the inset, it is shown the influence of static charge, *q*, on the relative stability, $\Delta E_2$, of AB-2 and $\hbox {AB}'$ with respect to AA-2.

**Fig. 3:**
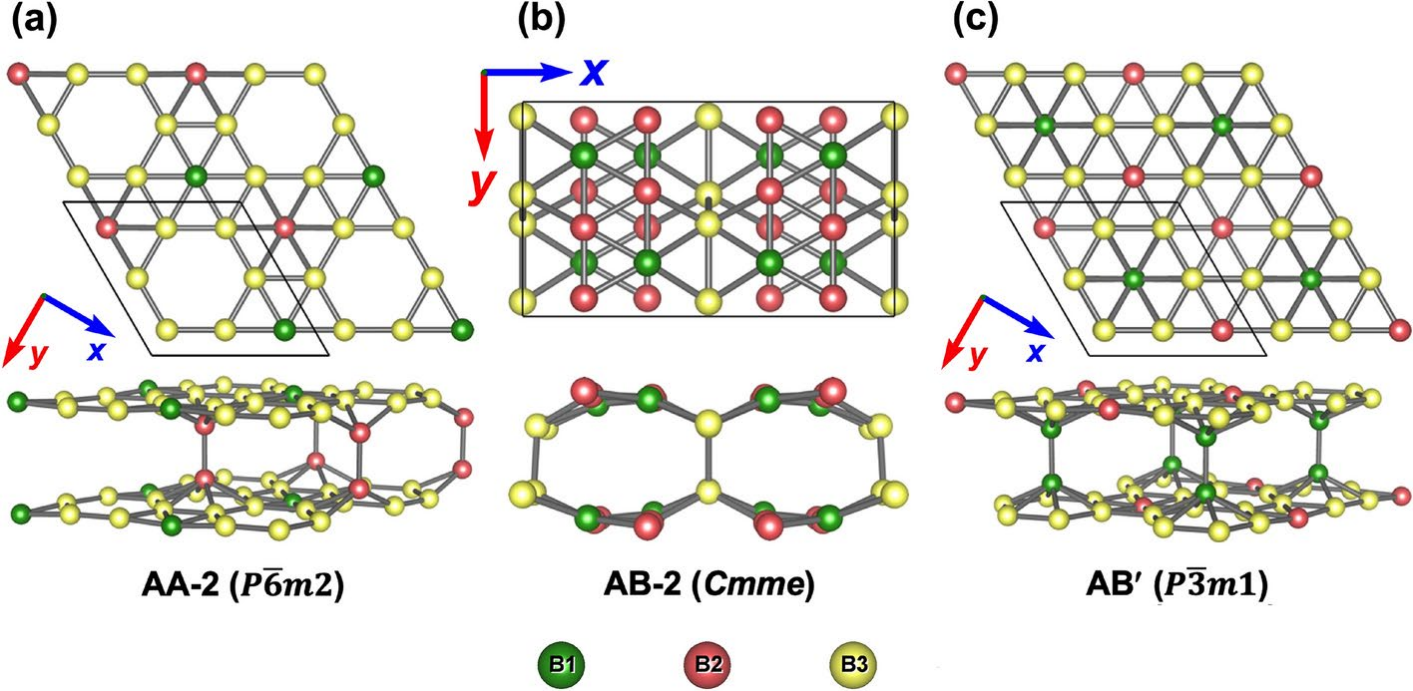
Top and side views of the (**a**) AA-2, (**b**) AB-2, and (**c**) $\hbox {AB}'$ structures. The B1, B2, and B3 labels of the boron atoms (for clarity, colored in green, red, and yellow, respectively) correspond to the atomic position listed in Table  1. In each case, the top view of the conventional unit cell is shown.

**Fig. 4:**
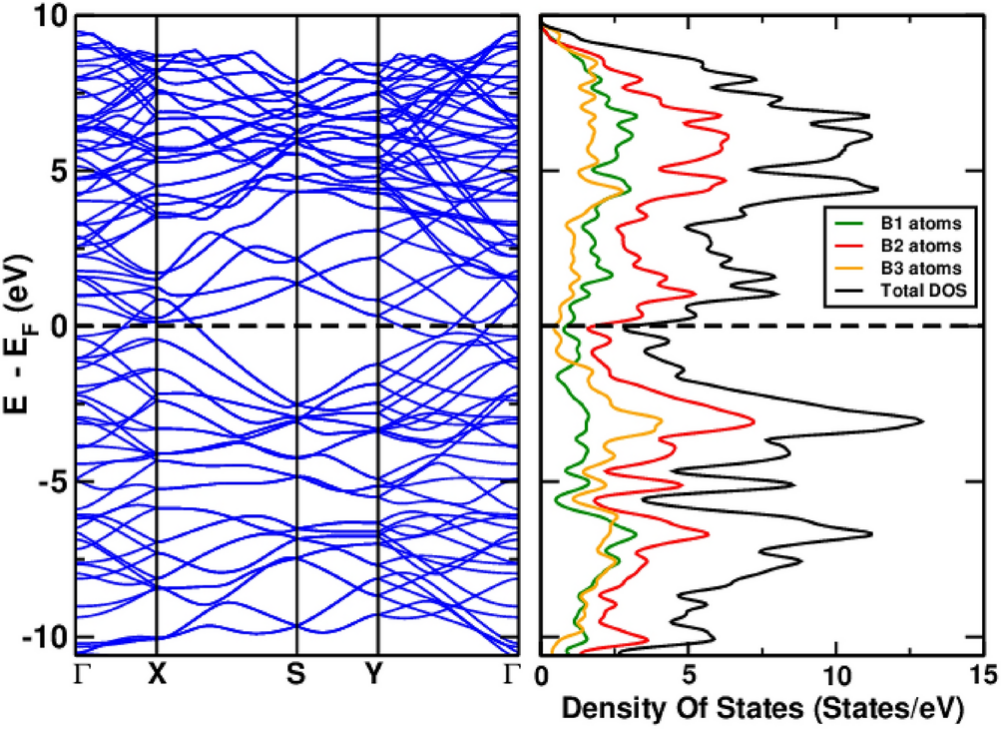
The electronic structure (left) and the projected density of states (right) for the AB-2 BL borophene.

**Fig. 5:**
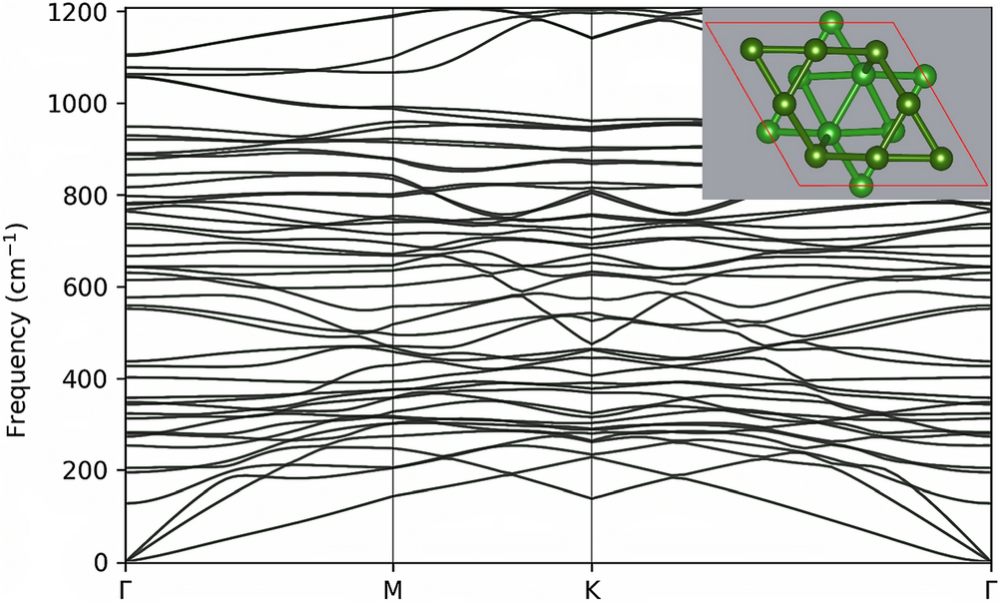
The phonon dispersion relation of AB-2 calculated for the reduced unit cell (16 atoms) shown in the inset.

**Fig. 6:**
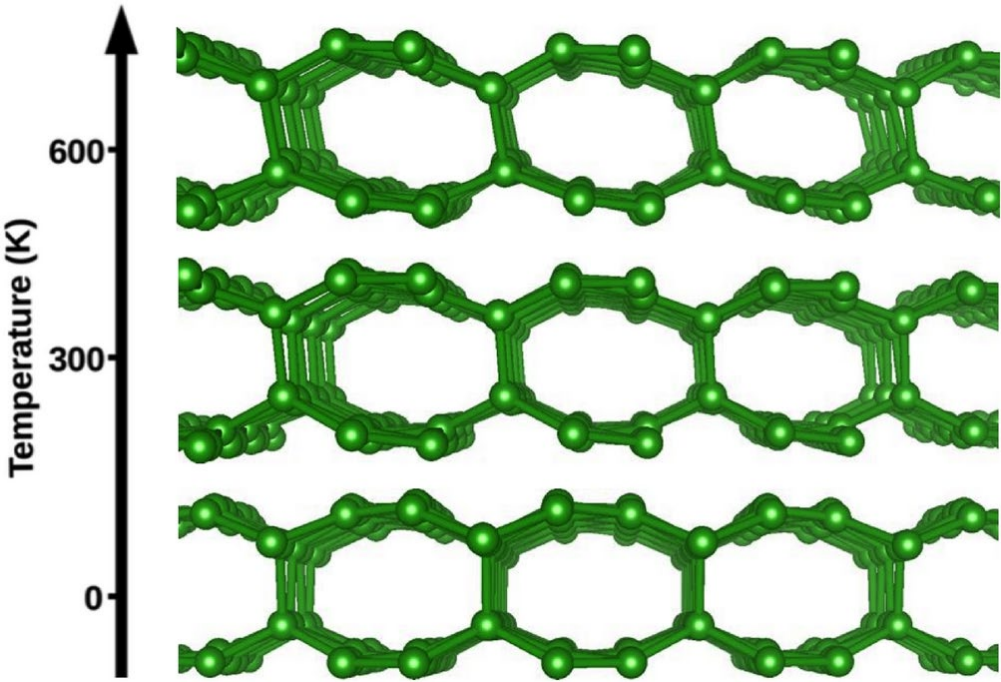
First-principles molecular dynamics simulations at 300 K and 600 K. The side view of the AB-2 structure is shown at each temperature.

Covalent bonds are formed between the layers if the borophenes are brought together to a distance smaller than $\sim 2$ Å. We show that two $\alpha$-sheets stacked directly on top of each other (AA-stacking) form chemical bonds when the smallest distance, *d*, between the layers is $\sim 1.8$ Å. The lowest energy structure for the AA-stacking is labeled in this work as AA-2 and is shown in Fig. 3a. The AA-2 structure has a single bond per unit cell that binds the $\alpha$-sheets together. This agrees with an earlier study confirming the AA-2 structure as the lowest in energy for the case of two $\alpha$-sheets brought together to a short distance in the AA configuration^20^. However, the experimentally obtained BL-$\alpha$ borophene, labeled in this work as AA-1, was described by having two covalent bonds per unit cell. This discrepancy may be attributed to the influence of the substrate on which the BL-$\alpha$ structure is supported^8^. For our freestanding case, the AA-2 structure is lower in energy than the AA-1 structure by 0.425 eV/atom. To understand better this large difference between the total energies of the two structures, we have calculated the energy dependence of the BL structure as a function of the distance between the composing atomic layers. This was done for the AA and AB stacking. The $\alpha$-sheets are brought together by gradually reducing the relative distance (in the *z* direction) between all the atoms. Structural optimization is performed at each step, but the *z* coordinates of the “floppy” atoms are fixed at a given distance. The results of these simulations are shown in Fig. 2. The AA-1 and AB-1 structures, shown in the insets of Fig. 2, having *P*6/*mmm* and *Cmme* symmetries, respectively, correspond to local minima in the total energy landscape. However, the AA-2 and AB-2 structures (shown in Fig. 3a and Fig. 3b, respectively), which are the results of structural optimization with no constraints of our initial configurations (shown in Fig. 1), are much lower in energy and have $P {\overline{6}} m 2$ and *Cmme* symmetries, respectively. As a complementary structure, we have added to this group the $\hbox {AB}'$ structure (shown in Fig. 3c), which is structurally similar to the AA-2 structure. Both structures have almost the same lattice constant, $a \approx 5.04$ Å, and one interlayer B–B bond per unit cell. However, $\hbox {AB}'$ exhibits a lower symmetry than AA-2 ($P {\overline{3}} m 1$ vs. $P {\overline{6}} m 2$, respectively). The $\hbox {AB}'$ structure has been previously studied theoretically^5^ and described as being similar to BL graphene. In Table 1, we collect the structural, energetic, and transport properties of the studied structures. As listed there, the AB-2 structure is energetically more favorable than the AA-2 structure by 20 meV/atom (23 meV/atom) in PBE (PBE-D3) calculations. The interlayer binding energy amounts to 15 meV/atom (41 meV/atom) for the AB-2 structure in PBE (PBE-D3) calculations. The shortest B–B distances between two opposing monolayers are 1.748, 1.663, and 1.749 Å for AA-2, AB-2, and $\hbox {AB}'$, respectively. All the studied BL structures are found to be of metallic nature and dynamically stable, which is shown for AB-2 in Figs. 4 and 5, respectively. This is consistent with previous reports for the AA-1 and $\hbox {AB}'$^5,8^ structures. To further verify the stability of AB-2, we have performed first-principles molecular dynamics (MD) simulations at two different temperatures, 300 and 600 Kelvin, and with the choice of 1 fs for the time step. Each run was 4.8 ps long. The AB-2 BL borophene does not undergo significant deformations at both temperatures, as shown in Fig. 6.

**Table 1:** Calculated lattice constants and the interlayer shortest distance (*d*), number of atoms per unit cell ($N_a$), number of interlayer B–B bonds per unit cell ($N_{\textrm{B}-\textrm{B}}$), atomic positions, cohesive energies ($E_c$), and conductivity components ($\sigma _{xx}$ and $\sigma _{yy}$) of the 2D boron structures. The values in brackets correspond to $E_c$ obtained within the dispersion-corrected DFT method (PBE-D3). The $\alpha$-sheet is buckled and has *C*2/*m* symmetry, however the buckling is small and the structure is often categorized as having *P*6/*mmm* symmetry.

Structure	$N_a$	$N_{\mathrm {B-B}}$	Symmetry	Lattice parameters (Å)	Atomic positions	Cohesive energy (eV/atom)	Conductivity ($\times 10^6~\mathrm {S/m}$)
$\alpha$-sheet	8	–	*C*2/*m*	$a = 8.76$ $b = 5.06$	B1 0.167 0.000 0.500 B2 0.166 0.334 0.500 B3 0.000 0.831 0.500	5.968 (5.998)	$\sigma _{xx} = 3.5$ $\sigma _{yy} = 3.5$
AA-1	16	2	*P*6/*mmm*	$a = 5.39$ $d = 1.81$	B1 0.333 0.667 0.440 B2 0.673 0.000 0.435	5.538 (5.590)	$\sigma _{xx} = 10.2$ $\sigma _{yy} = 10.1$
AA-2	16	1	$P {\overline{6}} m 2$	$a = 5.04$ $d = 1.75$	B1 0.333 0.667 0.382 B2 0.667 0.333 0.442 B3 0.333 0.003 0.390	5.963 (6.016)	$\sigma _{xx} = 7.6$ $\sigma _{yy} = 7.6$
AB-1	32	4	*Cmme*	$a = 10.41$ $b = 5.00$ $d = 1.60$	B1 0.898 0.000 0.555 B2 0.088 0.167 0.560 B3 0.250 0.675 0.553	5.675 (5.724)	$\sigma _{xx} = 18.7$ $\sigma _{yy} = 12.1$
AB-2	32	4	*Cmme*	$a = 8.65$ $b = 5.00$ $d = 1.66$	B1 0.165 0.250 0.405 B2 0.335 0.085 0.394 B3 0.000 0.069 0.445	5.983 (6.039)	$\sigma _{xx} = 4.3$ $\sigma _{yy} = 7.6$
$\hbox {AB}'$	16	1	$P {\overline{3}} m 1$	$a = 5.04$ $d = 1.75$	B1 0.000 0.000 0.442 B2 0.333 0.667 0.617 B3 0.003 0.670 0.389	5.961 (6.014)	$\sigma _{xx} = 6.8$ $\sigma _{yy} = 6.8$

To mimic the influence of the metallic substrate on structure of the BL borophenes, we study the relative stability of statically charged structures with charges ranging from 0 to 0.5 electrons per atom. In this range of charges, the experimentally observed AA-1 structure is energetically less stable than the AA-2 structure, suggesting that the preference for forming the AA-1 structure seen in the experiment is not purely of electrostatic nature. The AB-2, the most stable structure for zero charge, becomes less stable than AA-2 at charges larger than $\sim 0.05~e/\textrm{atom}$. This is shown in the top-right inset of Fig. 2. Interestingly, the $\hbox {AB}'$ and AA-2 structures exhibit virtually the same energy for the whole range of the studied charges.

Finally, we have studied the transport properties of the BL structures. The values for electric conductivity components, $\sigma _{xx}$ and $\sigma _{yy}$, are collected in Table  1. Except for the structures with 2-fold rotational symmetry (AB-1 and AB-2), all others have equal conductivity in the *x* and *y* directions. The projected density of states (PDOS) shown for AB-2 in Fig. 4 helps us to understand this result since the main contribution at the Fermi level comes from the B1 and B2 atoms shown in green and red in Fig. 3, respectively, which form boron double chains (BDC), all align in the *y* direction (see Fig. 3b). The highest conductivity is obtained for the AB-1 structure ($\sigma _{xx}=18.7 \times 10^6\mathrm {~S}/\textrm{m}$), whereas conductivity values as high as ${\sim } 10^7\mathrm {~S}/\textrm{m}$ are obtained for the experimentally observed AA-1 structure. For comparison, our value for bulk copper is $5.64 \times 10^7\mathrm {~S}/\textrm{m}$ in close agreement with the experimental value of $5.96 \times 10^7\mathrm {~S}/\textrm{m}$^21^.

## Summary

In summary, according to our calculations, the BL structures maintain the borophene’s desirable electronic properties (e.g., metallic behavior) while offering better stability. The presence of interlayer bonds reinforces the structural stability of the single layers. This is especially true for the AB-2 structure, which has a higher binding energy (regardless of the level of theory used) than two $\alpha$-sheets alone. This may be crucial for isolating the 2D structure from the substrate on which it would be grown. Finally, the experimentally obtained AA-stacking bilayer supported on a metallic substrate retains the 6-fold fold rotational symmetry of the $\alpha$-sheet. In contrast, the freestanding AB-stacking structure has 2-fold symmetry, which leads to the anisotropy in transport properties. The studied BL borophenes exhibit extraordinarily high electric conductivity with values as high as ${\sim } 10^7\mathrm {~S}/\textrm{m}$.

## Methods

First-principles calculations were performed within the framework of DFT using the PBE exchange-correlation functional and norm-conserving pseudopotentials^22^ as implemented in the Quantum ESPRESSO (QE) suite of codes^23^. To avoid interactions between the periodic replicas of the system, we considered an empty space of 15 Å (40 Å for the charged structures) thickness along the normal direction. Optimized geometries were reached allowing the unit cell shape, volume, and the ions to relax until the residual forces on the atoms were less than 0.3 meV/Å. The total energy ($E_t$) convergence was set to $10^{-5}$ Ry. We expanded the electronic wave functions in plane-wave basis sets with an energy cutoff of 80 Ry, while the $\Gamma$-centered Monkhorst–Pack *k*-point grid in the Brillouin zone was set to $12\times 12\times 1$ for the geometry optimization and $16\times 16\times 1$ for the phonon calculations. These values ensure the accuracy of $E_t$. The transport integrals have been computed using the Boltzmann transport theory^24^ and the constant scattering rate model (the inverse of relaxation time was taken to be $0.1~\textrm{eV}$). All the 2D crystal structures were drawn using the VESTA software^25^.

## Data Availability

The results of the present work are reported in the manuscript. Relevant data that support the findings of this study are available from the corresponding author upon reasonable request.
